# Accelerometer-derived physical activity and sedentary behaviors in individuals with newly diagnosed type 2 diabetes: A cross-sectional study from the Danish nationwide DD2 cohort

**DOI:** 10.3389/fspor.2022.1089579

**Published:** 2023-01-25

**Authors:** Sidsel L. Domazet, Jakob Tarp, Reimar W. Thomsen, Kurt Højlund, Jacob V. Stidsen, Jan C. Brønd, Anders Grøntved, Jens Steen Nielsen

**Affiliations:** ^1^ Steno Diabetes Center Odense, Odense University Hospital, Odense, Denmark; ^2^ Department of Clinical Research, University of Southern Denmark, Odense, Denmark; ^3^ Department of Clinical Epidemiology, Aarhus University, Aarhus, Denmark; ^4^ Department of Sports Science and Clinical Biomechanics, University of Southern Denmark, Odense, Denmark

**Keywords:** physical activity, type 2 diabetes, lifestyle and behaviour, sedentary activities, adults (MeSH)

## Abstract

**Introduction:**

Habitual physical activity behaviors of individuals with new-onset type 2 diabetes are largely unknown. We aimed to investigate accelerometer-derived physical activity behaviors in individuals with newly diagnosed type 2 diabetes. We also examined sociodemographic and health-related correlates of a high-risk physical activity profile.

**Methods:**

This cross-sectional study used data from 768 participants enrolled in an intervention study nested within the Danish Centre for Strategic Research in Type 2 diabetes (DD2) cohort. Physical activity was assessed by 24-h dual monitor accelerometry. Prevalence ratios of having a high-risk physical activity profile were estimated using Poisson regression adjusted for age and sex.

**Results:**

Study participants spent on average 9.7 (25th and 75th percentiles, 8.3; 11.1) hours/day sitting, walked for 1.1 (0.8; 1.6) hours/day and accumulated 4,000 (2,521; 5,864) steps/day. Still, 62% met the recommendations for physical activity. Characteristics associated with a high-risk physical activity profile (observed in 24.5% of participants) included older age, higher body mass index (BMI), unemployment, retirement, comorbidities, and current smoking. Hence, participants aged 60–69, 70–79 and 80+ years had prevalence ratios of 2.12 (95% CI 1.31; 3.42), 1.99 (1.18; 3.34) and 3.09 (1.42; 6.75) for a high-risk activity profile, respectively, versus participants <50 years. BMI values of 30–39 and 40+ were associated with 1.83 (1.06; 3.15) and 3.38 (1.88; 6.05) higher prevalence ratios compared to normal-weight. Unemployment or retirement was associated with 1.62 (1.09; 2.41) and 2.15 (1.37; 3.39) times higher prevalence ratios, compared to individuals in the working force. Having a Charlson Comorbidity Index score of 1–2 or 3+ was associated with 1.36 (1.03–1.79) and 1.90 (1.27–1.84) higher prevalence ratios, while current smoking was associated with a prevalence ratio of 1.72 (1.25; 2.35) compared to never smokers.

**Conclusion:**

This study shows that 62% of individuals with newly diagnosed type 2 diabetes met the recommendations for physical activity. Still, the majority of participants were also highly sedentary and accumulated very few daily steps, emphasizing the need for focusing on both increasing physical activity and reducing sedentary behaviors in the prevention of diabetes-related complications. Individuals with a high-risk physical activity profile were characterized by more obesity, socioeconomic inequalities, advanced age and comorbidities.

Trial registration number: NCT02015130.

## Introduction

Individuals with type 2 diabetes have a higher risk of cardiovascular disease (CVD) events and mortality even when risk factors are optimally managed (1, 2). Regular physical activity may abate this risk. However, more than a quarter of the world's adult population are insufficiently physically active, according to recommended levels of moderate-to-vigorous physical activity for prevention and management of non-communicable diseases (3). Additionally, individuals with type 2 diabetes have a lower physical activity level compared to age-matched individuals without chronic conditions in the UK Biobank cohort (4). Data from the US Behavioral Risk Factor Surveillance System survey have further suggested that only 40% of individuals with prevalent type 2 diabetes achieve recommended levels of moderate-to-vigorous physical activity for the prevention of diabetes-related complications (5).

Recently, the evidence supporting health-related benefits of physical activity has expanded from focusing on moderate-to-vigorous physical activity to include the total volume of activity, including light physical activity, and to limit the amount of time spent sedentary (6, 7). This development is supported by body-mounted devices that provide a real-time assessment of habitual physical activity behaviors while bypassing cognitive biases related to self-report. Reducing device-measured sedentary behavior has been shown to improve insulin sensitivity (8), postprandial glycemic control and lipid metabolism (9–11). Sedentary time has further been independently associated with mortality risk (12). This evidence highlights the importance of considering the whole spectrum of physical activity and including sedentary time as an independent target to achieve similar health benefits (6). Engagement in moderate-to-vigorous physical activity is potentially an unfeasible target for lifestyle intervention in individuals with type 2 diabetes (13), while a new potential target might be to limit sedentary time by promoting lower intensity activities of the daily living (i.e., active transportation or less sitting).

There are currently no comprehensive analyses evaluating habitual physical activity behaviors in new-onset type 2 diabetes, which is essential to guide development of feasible physical activity programs at a point in time, where the potential for prevention of vascular complications and mortality is greatest. We therefore aimed to comprehensively investigate accelerometer-derived habitual physical activity volume, intensity and behaviors in individuals with newly diagnosed type 2 diabetes. We also investigated which sociodemographic and health-related factors correlated with a high-risk physical activity profile defined by high sedentary time in combination with low moderate-to-vigorous physical activity.

## Materials and methods

### Sampling

**Figure 1:**
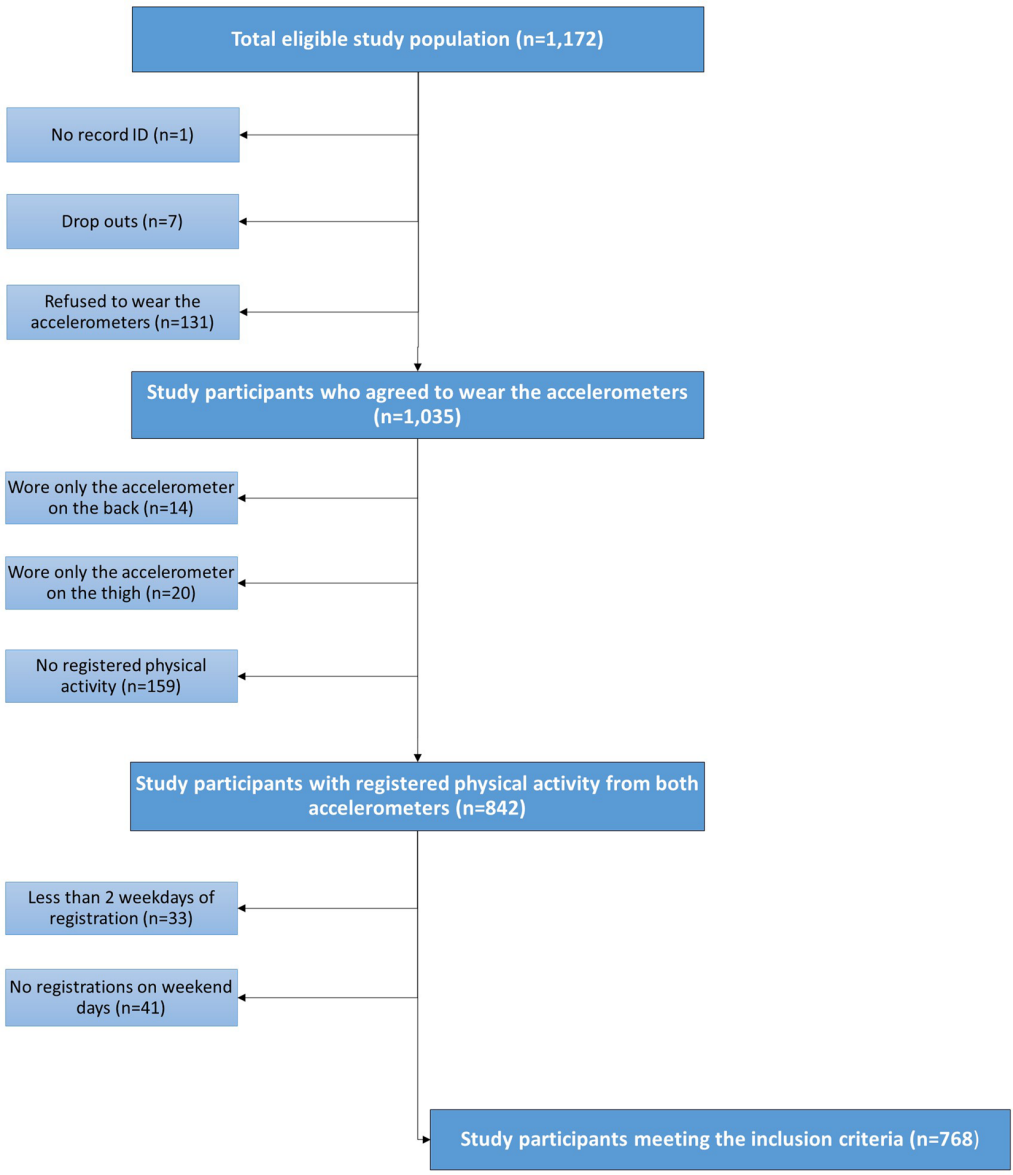
Flow diagram of study participants in IDA with registered physical activity and sedentary time.

This cross-sectional study used baseline data from a prospective controlled multicenter open-label intervention study—The Specialist Supervised Individualized Treatment of New Clinically Diagnosed Type 2 Diabetes in General Practice (IDA) (14). A total of 1,172 individuals with type 2 diabetes gave written informed consent to participate in IDA. The participants were recruited among patients with new-onset type 2 diabetes (median 3.5 years), who were enrolled in a nationwide patient cohort—The Danish Centre for Strategic Research in Type 2 diabetes (DD2) (15). A flow diagram of study participants is presented in Figure 1. IDA collected baseline data from 2013 to 2018 of which individuals were eligible for inclusion provided that they (1) were followed at a general practitioner participating in DD2, (2) unlikely had type 1 diabetes (defined as age <30 years at DD2 enrollment, fasting C-peptide <300 pM and GAD 65-ab >20 IU/ml), (3) had a life expectancy >2 years, and (4) did not participate in other clinical trials than IDA. The mean time between enrollment in DD2 and baseline examination in IDA was 1.3 years (SD 1.7 years). The IDA study has been approved by the Regional Committee on Medical Health Ethics (Region of Southern Denmark S-20120186), the Danish Data Protection Agency and Medicines Authority (journal no. 2012120204). The study was conducted in concordance with the Helsinki declaration II.

### Outcome: Physical activity patterns

Physical activity including sedentary behaviors, sleep and step count were assessed by 24-h dual monitor accelerometry. On the test day, two tri-axial accelerometers (Axivity AX3, Axivity, Newcastle, UK) were attached directly on the skin with waterproof tape. One accelerometer was placed on the lower back to capture physical activity volume and intensity, while another was placed on the right thigh mainly to capture step count and movement behaviors based on the acceleration signal and the thigh inclination (16, 17). The study participants were instructed to wear both accelerometers continuously for 10 days including during showering or any other water activities. Inclusion criteria for a valid physical activity registration were defined as wearing the accelerometer with (1) ≥22 h of daily wear time, (2) on ≥2 weekdays (Mon-Fri) and (3) on ≥1 weekend day (Sat-Sun). A total of 1,172 participants were eligible, whereof 332, 33, and 41 participants did not comply with criteria 1, 2, and 3, respectively (Figure 1). Observations below 3 standard deviations from the mean for sedentary time, light physical activity, and physical activity behaviors of lying, sitting, standing, moving and walking were further excluded. This procedure did not change the final number of observations as the inclusion criteria was still met. For more detailed information of the setup we refer to the study protocol (14).

Physical activity intensities were determined as time spent in the following domains; sedentary, light, moderate and vigorous using ActiGraph® counts generated from raw acceleration measured at the back using an epoch length of 10 s (18). Moderate-to-vigorous physical activity were based on age-specific cut points determined by the average intensity in counts at preferred walking speed for moderate and running equivalent to 60% of VO_2_ max for vigorous. The cut points were derived from an internally conducted calibration study using established methods (19). Cut points are reported in Supplementary Material 1. Sedentary time was defined as registrations below 100 counts/minute (20). Physical activity behaviors were determined as time spent; lying, sitting, standing, moving, walking, biking and running using the classification algorithm based on raw acceleration proposed by Skotte et al. (17). The classification of physical activity behaviors has been evaluated using a standardized field test showing sensitivity and specificity above 95.3% for all included activities. The behavior “moving” covers minor movement while standing (i.e., dishwashing) and captures activities that remain after classification of lying, sitting, standing and walking. As 24-h registration allows for assessment of sleep, we deduced sleep from the last recorded activity registration before midnight (00:00) including uninterrupted sedentary behavior in an inclined body position until the first activity registration after 6:00 AM the next day. To account for possible differences in daily physical activity level between weekdays and weekends, all physical activity estimates were presented as a weighted average according to 5/7 (Monday-Friday) and 2/7 (Saturday-Sunday).

### Exposures: Sociodemographic and health-related factors

Information on age and sex was derived from participants’ civil registration number, which also facilitated linkage with Danish registries (21, 22). Body weight and height were measured by standard anthropometric procedures on the test day and used to calculate body mass index (BMI) (kg/m^2^). Sociodemographic factors including education level and work status as well as smoking status were gathered *via* a questionnaire completed on the test day. Education level was divided into 3 categories (1) primary school/elementary school (75%) or secondary school/high school/trainee, (2) medium-term education (i.e., nurse, craft worker, teacher) and (3) long-term education (academic). Work status was likewise divided into 3 categories comprising (1) Employment including part-time employment (17%), (2) retirement including early retirement (13%) and (3) unemployment including sick leave (17%) and early retirement due to disability (57%). Smoking status was defined as never, former or current smoker including occasional smoking (9%) according to self-reported tobacco consumption. Blood pressure was assessed by automated ambulatory blood pressure every 3rd minute for 30 min using the Mobil-O-Graph system (IEM, Stolberg, Germany). The average of the repeated measurements represented the final systolic and diastolic blood pressure. Biochemical measures of hemoglobin A_1_c (HbA_1_c), LDL-cholesterol and creatinine were measured in routine care from a blood sample collected by the general practitioner or hospital closest to study enrollment. From fasting plasma samples glucose and serum C-peptide were measured and indices of beta-cell function and insulin sensitivity were calculated according to the Homeostatic Model Assessment (HOMA2) (23). The estimated glomerular filtration rate (eGFR) was calculated from serum creatinine using the CKD-EPI Equation for males and females, separately (24, 25). Information on prescribed medication was collected from The Danish National Prescription Registry containing all prescribed drug dispensations from Danish pharmacies since 1994. Information on existing comorbidities were gathered from The Danish National Patient Register. The total burden and severity of comorbidities were calculated according to the Charlson Comorbidity Index (CCI), excluding diabetes and presented categorically (0, 1–2, and 3+) (26). Records of both comorbidities and drug prescriptions were tracked 10 years back from study enrollment (index date). Diabetes duration was calculated from the number of days between diabetes diagnosis and the test day. Diagnosis of diabetes was obtained from the DD2 interview or otherwise defined from the first occurring event of the following (1) first glucose-lowering drug prescription, (2) first diabetes-related contact to the hospital system, (3) first registration in the Danish Adult Diabetes Registry or (4) study enrollment in DD2.

### Statistics

Clinical characteristics of the study participants are displayed in medians with interquartile intervals (IQI) or in numbers with percentage for count data. Crude estimation of accelerometer compliance, physical activity level and behaviors of study participants are also displayed in medians with IQI.

To identify sociodemographic and health-related factors associated with a high-risk physical activity profile, the study sample was divided into four mutually exclusive groups from combinations of high/low sedentary time and moderate-to-vigorous physical activity according to a median split for sedentary time (median 648 min/day) and meeting/not-meeting the recommended levels of 30 min/day moderate-to-vigorous physical activity according to the Danish Health Authorities (27). The high-risk physical activity profile was defined as high sedentary time (>648 min/day) and low moderate-to-vigorous physical activity (<30 min/day), whereas the opposite combination of low sedentary time and high moderate-to-vigorous physical activity was most favorable. Prevalence ratios of having a high-risk physical activity profile by sociodemographic and health-related factors were estimated using Poisson regressions with robust standard errors. In our main model, we controlled for age and sex (model 1). We refrained from further confounder adjustments in our main analysis, because many of the sociodemographic and health-related factors do not qualify as confounders, since they are bidirectionally associated with each other and may act as intermediates and clusters in the same pathophysiological pathways. As an example, low education, smoking and high BMI may cause comorbidities and may thereby in turn decrease physical activity, but primary comorbidities may also decrease education level and alter smoking habits and BMI on the way towards physical activity. To explore if associations between individual sociodemographic and health-related factors depended on the other factors, we did an additional analysis (model 2), where prevalence ratios were mutually adjusted for remaining exposures to determine the association among interrelated factors.

All results derived from complete case analysis. Assessment of model fit was performed by goodness-of-fit chi-squared test and residuals were visually inspected for heteroscedasticity. All model assumptions were fulfilled with no obvious heteroscedasticity of residuals. An alpha of 0.05 was used as the threshold for statistical significance. All statistical analyses were carried out in Stata/BE 17.0 (StataCorp LLC, Texas, US).

## Results

From the total eligible sample (*n* = 1,172), 1,035 study participants agreed to wear the accelerometers of which 768 participants (74%) fulfilled the inclusion criteria for a valid activity registration period (Figure 1). Characteristics of the final study population stratified by physical activity profile are displayed in Table 1. Compared to the other groups, individuals with a high-risk physical activity profile were on average older, more obese, had a higher C-peptide level and insulin secretion but lower insulin sensitivity and estimated glomerular filtration rate (Table 1).

**Table 1:** Characteristics of study participants stratified by physical activity profiles.

	Total	Low sedentary/high MVPA	High sedentary/high MVPA	Low sedentary/low MVPA	High sedentary/low MVPA
		Median (IQI)	Median (IQI)	Median (IQI)	Median (IQI)
*N*, (%)	768 (100)	280 (36.5)	197 (25.7)	103 (13.4)	188 (24.5)
Females, *n* (%)	323 (42.1)	108 (38.6)	64 (32.5)	57 (55.3)	94 (50.0)
Age, years	61.8 (53.7; 68.5)	58.6 (51.6; 65.5)	61.2 (52.9; 69.0)	64.1 (55.7; 72.3)	65.6 (56.9; 69.6)
Diabetes duration, years	3.5 (0.9; 5.9)	3.3 (0.9; 6.0)	3.4 (0.8; 5.8)	4.0 (1.1; 6.3)	3.3 (0.8; 5.6)
Height, cm	172 (165; 178)	173 (165; 179)	174 (167; 178)	167 (162; 176)	170 (164; 176)
Weight, kg	91.0 (80.1; 105.2)	89.0 (79.0; 99.6)	93.8 (82.3; 108.6)	87.6 (77.7; 102.0)	96.6 (83.5; 109.8)
BMI, kg/m^2^	31.0 (28.0; 34.9)	29.7 (27.2; 32.8)	31.0 (28.1; 35.8)	31.2 (27.7; 35.2)	33.1 (29.6; 36.8)
Systolic BP, mmHg	127 (120; 136)	128 (121; 136)	128 (120; 136)	125 (118; 133)	127 (119; 137)
Diastolic BP, mmHg	81 (75; 88)	84 (77; 89)	81 (74; 88)	80 (74; 85)	80 (73; 87)
HbA1c, mmol/mol	49 (45; 55)	49 (45; 55)	49 (45; 55)	50 (45; 55)	48 (45; 55)
HbA1c, %	6.6 (6.3; 7.2)	6.6 (6.3; 7.2)	6.6 (6.3; 7.2)	6.7 (6.3; 7.2)	6.5 (6.3; 7.2)
C-peptide, pmol/L	1,123 (862; 1,504)	1,062 (828; 1,367)	1,039 (810; 1,330)	1,217 (892; 1,575)	1,323 (975; 1,710)
HOMA-beta, %	83.8 (62.6; 111.7)	77.6 (59.3; 101.2)	79.8 (59.4; 105.1)	84.7 (65.4; 104.9)	100.4 (73.1; 129.7)
HOMA-S, %	35.1 (26.0; 45.8)	37.4 (28.7; 47.5)	38.1 (29.3; 48.8)	32.5 (25.7; 43.4)	29.7 (21.9; 41.4)
LDL-cholesterol, mmol/L	2.1 (1.6; 2.7)	2.1 (1.6; 2.8)	2.1 (1.6; 2.6)	2.1 (1.7; 2.8)	2.1 (1.6; 2.6)
eGFR, ml/min/1.73^2^	88.6 (76.3; 98.2)	91.9 (81.0; 100.3)	88.1 (76.3; 97.1)	89.5 (72.0; 97.1)	83.1 (69.3; 94.3)
Glucose-lowering drugs, *n* (%)	607 (79.0)	224 (80.0)	143 (72.6)	84 (81.6)	156 (83.0)
Insulin, *n* (%)	25 (3.3)	5 (1.8)	9 (4.6)	5 (4.9)	6 (3.2)
Lipid-lowering drugs, *n* (%)	502 (65.4)	173 (61.8)	135 (68.5)	71 (68.9)	123 (65.4)
Anti-hypertensive drugs, *n* (%)	494 (64.3)	169 (60.4)	118 (59.9)	72 (69.9)	135 (71.8)

IQI, interquartile intervals (25th and 75th percentiles). MVPA, moderate-to-vigorous physical activity. Low sedentary, ≤648 min/day. High MVPA, ≥30 min/day. High sedentary, >648 min/day. Low MVPA, <30 min/day. High-risk physical activity profile; high sedentary/low MVPA. BP, blood pressure; eGFR, estimated glomerular filtration rate.

### Physical activity patterns

Crude estimates of accelerometer compliance and daily physical activity level and behaviors stratified by physical activity profiles are displayed in Table 2. Overall, study participants spent on average 9.7 (IQI 8.3; 11.1) hours/day sitting, walked for 1.1 (IQI 0.8; 1.6) hours/day and accumulated 4,000 (IQI 2,521; 5,864) steps/day. Biking and running were rare (≤1 min/day). Of the total study population, 62% met recommended levels of 30 min/day moderate-to-vigorous physical activity equal to an overall daily average of 28 min (IQI 18; 41) of moderate and 6 min (IQI 2; 14) of vigorous physical activity. Stratification into physical activity profiles showed a gradual decline in daily physical activity level from the most favorable physical activity profile to the high-risk physical activity profile. Hence participants with a high-risk physical activity profile took less than half as many steps (2,152 steps/day vs. 5,510 steps/day), walked less than half as much (39 min/day vs. 93 min/day) and spent 73% less time on moderate-to-vigorous physical activity (14 min/day vs. 52 min/day) compared to participants with the most favorable physical activity profile. At the same time, sedentary time and time spent sitting were higher by 2.4 h/day and 2.9 h/day for participants with a high-risk physical activity profile.

**Table 2:** Crude median estimation of physical activity volumes, intensities and behaviors in individuals with newly diagnosed type 2 diabetes stratified by physical activity profiles.

	Total	Low sedentary/high MVPA	High sedentary/high MVPA	Low sedentary/low MVPA	High sedentary/low MVPA
		Median (IQI)	Median (IQI)	Median (IQI)	Median (IQI)
*N*, %	768 (100)	280 (36.5)	197 (25.7)	103 (13.4)	188 (24.5)
Accelerometer compliance					
Number of days, days	9 (8; 10)	9 (8; 10)	9 (9; 10)	8 (6; 9)	9 (8; 10)
Awake time, min/day	885 (827; 929)	859 (809; 902)	925 (896; 957)	789 (751; 832)	905 (865; 939)
Non-wear time, min/day	0 (0; 0)	0 (0; 0)	0 (0; 0)	0 (0; 0)	0 (0; 0)
Overall physical activity level					
Steps/day	4,000 (2,521; 5,864)	5,510 (4,194; 7,371)	4,651 (3,705; 6,179)	2,573 (1,731; 3,458)	2,152 (1,321; 3,184)
Physical activity intensities					
Sedentary, min/day	648 (592; 707)	590 (538; 620)	692 (668; 726)	603 (560; 626)	729 (691; 768)
Light, min/day	185 (147; 220)	218 (182; 247)	177 (148; 208)	184 (147; 216)	146 (122; 180)
Moderate, min/day	28 (18; 41)	39 (31; 53)	34 (27; 43)	19 (12; 22)	13 (8; 20)
Vigorous, min/day	6 (2; 14)	13 (6; 23)	11 (6; 19)	2 (1; 3)	1 (1; 3)
Physical activity behaviors					
Sleep, min/day	486 (443; 542)	514 (469; 560)	466 (419; 495)	559 (499; 618)	455 (416; 499)
Lying, min/day	492 (434; 550)	519 (473; 572)	460 (407; 498)	580 (516; 653)	452 (397; 500)
Sitting, min/day	581 (496; 664)	501 (449; 561)	636 (585; 680)	522 (462; 585)	678 (612; 747)
Standing, min/day	198 (148; 255)	221 (166; 278)	194 (152; 243)	185 (140; 253)	174 (119; 242)
Moving, min/day	37 (23; 52)	45 (32; 63)	36 (26; 47)	38 (23; 53)	24 (15; 37)
Walking, min/day	67 (47; 93)	93 (71; 117)	72 (59; 88)	48 (36; 61)	39 (28; 52)
Biking, min/day	1 (0; 5)	2 (0; 10)	1 (0; 7)	0 (0; 2)	0 (0; 2)
Running, min/day	0 (0; 0)	0 (0; 0)	0 (0; 0)	0 (0; 0)	0 (0; 0)

IQI, interquartile intervals (25th and 75th percentiles). MVPA, moderate-to-vigorous physical activity. Low sedentary, ≤648 min/day. High MVPA, ≥30 min/day. High sedentary, >648 min/day. Low MVPA, <30 min/day. High-risk physical activity profile; high sedentary/low MVPA. Non-wear time; time where at least one of the accelerometers has been detached from the body.

Since very few study participants engaged in running activities, the study population average was equal to zero. When we restricted our analysis to study participants who engaged in running for at least 2 min/day, our sample was reduced to 20 individuals (2.6%) with a median of 4 min/day (IQI 2; 7). We did the same for biking and found a median time of 8 min/day (IQI 4; 18) among 289 individuals (37.6%).

### High-risk physical activity profile

Cross tabulation was performed to quantitatively analyze the frequency and distribution of a high-risk physical activity profile across sociodemographic and health-related factors (Table 3). The most frequent physical activity profile was the most favorable (36.5%). Hence a combination of low sedentary time and high moderate-to-vigorous physical activity was most frequent among study participants of both sexes (females = 33.4%, males = 38.7%), who were under the age of 70 years (<50 years = 48.7%, 50–59 years = 43.5%, 60–69 years = 33.0%), with a BMI under 40 kg/m^2^ (BMI <25 = 43.8%, BMI 25–29.9 = 45.7%, BMI 30–39.9 = 33.1%), with a short (32.5%) or medium (40.4%) term education, who were working (46.4%) or retired (33.0%), without comorbidities (40.6%), and who had never (40.5%) or formerly (35.3%) smoked. A physical activity profile of both low sedentary time and low moderate-to-vigorous physical activity was least frequent (13.4%), whereas a physical activity profile of both high sedentary time and high moderate-to-vigorous physical activity was most frequent among higher educated study participants (long term education 37.5%). A quarter of the study participants presented with a high-risk physical activity profile (24.5%), which was dominated by older age (80 + years = 42.8%), higher BMI (40 + kg/m^2^ = 43.4%), unemployment (including sick leave and early retirement due to disability) (33.8%), comorbidities (1–2 = 30.8%, 3+ = 50.0%) and current smoking (33.6%).

**Table 3:** Proportions of study participants within each strata of sociodemographic and health-related factors stratified by physical activity profiles.

	Low sedentary/high MVPA	High sedentary/high MVPA	Low sedentary/low MVPA	High sedentary/low MVPA
*N* (%)	280 (36.5)	197 (25.7)	103 (13.4)	188 (24.5)
Sex				
Females	108 (33.4)	64 (19.8)	57 (17.7)	94 (29.1)
Males	172 (38.7)	133 (29.9)	46 (10.3)	94 (21.1)
Age				
<50	58 (48.7)	31 (26.1)	13 (10.9)	17 (14.3)
50–59	94 (43.5)	57 (26.4)	23 (10.7)	42 (19.4)
60–69	94 (33.0)	69 (24.2)	37 (13.0)	85 (29.8)
70–79	34 (25.4)	36 (26.9)	26 (19.4)	38 (28.3)
80+	0 (0.0)	4 (28.6)	4 (28.6)	6 (42.8)
BMI				
<25	28 (43.8)	15 (23.4)	10 (15.6)	11 (17.2)
25–29.9	117 (45.7)	66 (25.8)	31 (12.1)	42 (16.4)
30–39.9	123 (33.1)	94 (25.3)	53 (14.2)	102 (27.4)
40+	12 (15.8)	22 (29.0)	9 (11.8)	33 (43.4)
Education level				
Short term	109 (32.5)	72 (21.5)	54 (16.1)	100 (29.9)
Medium term	154 (40.4)	107 (28.1)	43 (11.3)	77 (20.2)
Long term	14 (29.2)	18 (37.5)	6 (12.5)	10 (20.8)
Work status				
Working	156 (46.4)	106 (31.5)	23 (6.9)	51 (15.2)
Unemployed	84 (27.0)	74 (23.8)	48 (15.4)	105 (33.8)
Retired	40 (33.0)	17 (14.0)	32 (26.5)	32 (26.5)
Comorbidities				
0	219 (40.6)	144 (26.7)	65 (12.0)	112 (20.7)
1–2	57 (28.8)	49 (24.7)	31 (15.7)	61 (30.8)
3+	4 (13.3)	4 (13.3)	7 (23.3)	15 (50.0)
Smoking				
Never	128 (40.5)	91 (28.8)	34 (10.8)	63 (19.9)
Former	110 (35.3)	77 (24.7)	47 (15.0)	78 (25.0)
Current	42 (30.0)	29 (20.7)	22 (15.7)	47 (33.6)

MVPA, moderate-to-vigorous physical activity. Low sedentary, ≤648 min/day. High MVPA, ≥30 min/day. High sedentary, >648 min/day. Low MVPA, <30 min/day. High-risk physical activity profile; high sedentary/low MVPA.

**Figure 2:**
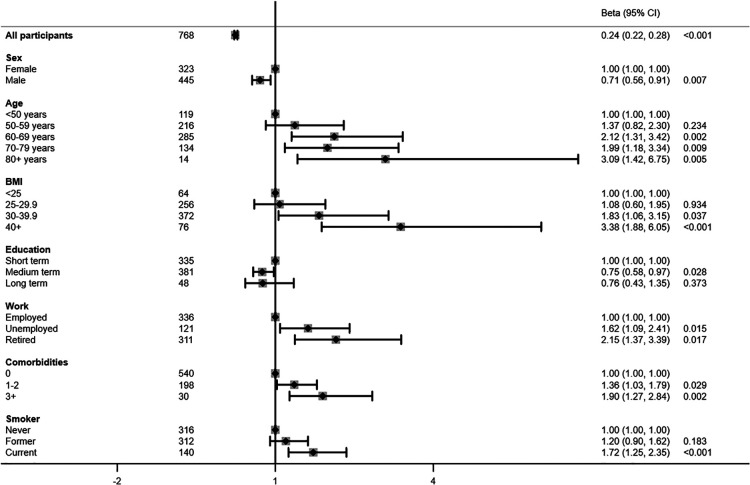
Risk factors associated with a high-risk physical activity profile presented in prevalence rate ratios adjusted for age and sex.

**Figure 3:**
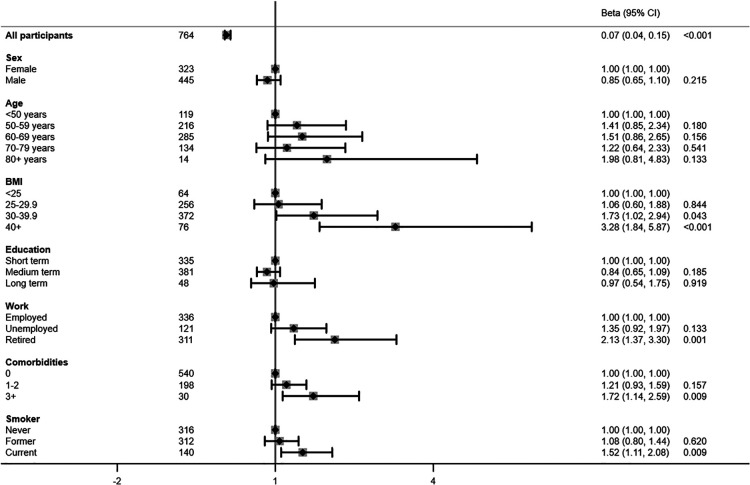
Risk factors associated with a high-risk physical activity profile presented in prevalence rate ratios mutually adjusted for all other risk factors.

The above-mentioned risk factors were also associated with higher prevalence ratios (PR) of a high-risk physical activity profile when adjusted for age and sex (Figure 2). Hence the PR of a high-risk physical activity profile was 2-fold higher among study participants between age 60–69 years (95% CI 1.31; 3.42) and 70–79 years (95% CI 1.18; 3.34), and was more than 3-fold higher in those aged 80 years or older (95% CI 1.42; 6.75), compared with participants aged younger than 50 years. Study participants with a BMI of 30–39 kg/m^2^ had a 1.8-fold higher prevalence of a high-risk physical activity profile (95% CI 1.06; 3.15), while the prevalence was 3.4-fold higher among those with a BMI of 40 kg/m^2^ or higher (95% CI 1.88; 6.05). Both unemployment (PR 1.62, 95% CI 1.09; 2.41) and retirement (PR 2.15, 95% CI 1.37; 3.39) were likewise risk factors for a high-risk physical activity profile. Comorbid conditions were associated with 1.36 (CCI 1–2 = 95% CI 1.03; 1.79) and 1.90 (CCI 3+ = 95% 1.27; 2.84) times higher PR of a high-risk physical activity profile. Current smoking was also a clear risk factor, displayed by a 1.72-fold higher prevalence of a high-risk physical activity profile (95% CI 1.25; 2.35). On the contrary, male sex (PR 0.71, 95% CI 0.56; 0.91) and medium (PR 0.75, 95% CI 0.58; 0.97) or long term education (PR 0.76, 95% CI 0.43; 1.35) were associated with a reduced prevalence of a high-risk physical activity profile. Most associations remained after mutual adjustment, although associations with age and education level attenuated (Figure 3).

## Discussion

The majority of individuals with newly diagnosed type 2 diabetes were both meeting the moderate-to-vigorous physical activity guidelines and spending large amounts of time on sedentary activities including sitting for almost 10 h/day. On average study participants only accumulated 4,000 steps/day. In adults with newly diagnosed type 2 diabetes, old age, obesity, unemployment, retirement, prevalent comorbidities and current smoking were correlates of a particularly unfavorable physical activity profile with high sedentary time and low moderate-to-vigorous physical activity. These results emphasize the importance and need for duality in physical activity goal setting in individuals with type 2 diabetes, as reducing sedentary time and promoting moderate-to-vigorous physical activity may be equally important targets.

Even though there is no established recommendations on the exact amount of daily sedentary time, evidence suggests that the risk of all-cause and CVD mortality increases when sedentary time exceeds 9–10 h/day (28). It is currently up to debate whether higher levels of moderate-to-vigorous physical activity can mitigate the mortality risk associated with higher levels of sedentary behaviors (29–31). A harmonized meta-analysis has concluded that about 30–40 min of moderate-to-vigorous physical activity per day attenuate the association between sedentary time and risk of death. However, when the association between sedentary time and mortality risk was analyzed within each strata of physical activity (low/medium/high) instead of being compared to the best possible reference (high moderate-to-vigorous physical activity and low sedentary), sedentary time was independently associated with greater mortality risk (30). This debate emphasizes the importance of targeting sedentary time as an independent risk factor that can co-exist with a high moderate-to-vigorous physical activity level.

In comparison with population-based cohorts of similar age, our study population had a low total volume of physical activity and took fewer steps (32–34). Hence the absolute volume of moderate-to-vigorous physical activity was higher among participants in The Copenhagen City Heart Study (CCHS) (70 min/day) and the Swedish CArdioPulmonary bio-Image Study (SCAPIS) (49 min/day) (34, 35). Previous studies have suggested that individuals with type 2 diabetes are less physically active compared to background populations (4, 36) and that many do not meet the guidelines for moderate-to-vigorous physical activity (5). The majority of our study population engaged in recommended levels of moderate-to-vigorous physical activity while their average sedentary time exceeded 10 h/day. The same trend recurred in the CCHS that also reported high levels of moderate-to-vigorous physical activity (70 min/day) in combination with high levels of sedentary behaviors (9.7 h/day) (34). SCAPIS on the other hand only reported 7.9 h of daily sedentary time. Although, 28% of the middle-aged participants in SCAPIS were defined as having an “at risk” physical activity behavior, which included high sedentary time (≥9.5 h per day) and low vigorous physical activity (<75 min per week) (35). However, general differences in processing of accelerometer-derived physical activity in terms of selection of intensity cut points, algorithm and inclusion criteria limit a head-to-head comparison of absolute physical activity levels across studies. In summary, these comparisons stress the importance of acknowledging the whole spectrum of physical activity and that high sedentary time can co-exist with a high moderate-to-vigorous physical activity level.

We found older age, higher BMI, unemployment, retirement, comorbidities and current smoking to be associated with a high-risk physical activity profile consisting of high sedentary time and low moderate-to-vigorous physical activity. Another study on correlates of physical activity in individuals with type 2 diabetes has reported similar associations in addition to higher perceived stress and lower health-related quality of life being negatively associated with physical activity levels (37). Our results showed that especially the presence of more than two comorbid conditions was associated with an almost 2-fold higher prevalence of a high-risk physical activity profile. This supports evidence from the UK Biobank (4). Surprisingly, we discovered current smoking as an independent risk factor for a high-risk physical activity profile probably due to clustering of risk factors. For example, the associations of age and education with a high-risk physical activity profile were blunted in the mutual-adjusted model, which may indicate that BMI, comorbidity and smoking mediated these associations. Current smokers and participants with BMI ≥30 were younger, whereas participants with comorbid conditions were substantially older (Supplementary Material 2). Having a short term education was also more prevalent among current smokers (54.7%) and participants with BMI ≥30 (47%) and >2 comorbid conditions (53.3%) (Supplementary Material 2). Since higher BMI was a consistent correlate of low physical activity, obese individuals with newly diagnosed type 2 diabetes may be a specific target for future physical activity interventions. Importantly, a higher level of physical activity has been associated with lower risk of mortality irrespective of weight status in a population-based study (38), and weight status *per se* predicts inactivity more than type 2 diabetes does (39). Physical activity has further been found to improve healthy weight loss maintenance in non-diabetic obese adults more than drug therapy or exercise alone (40).

One of the major strengths of this study is the unique cohort of individuals with clinically diagnosed new-onset type 2 diabetes followed in the primary sector. The final study sample is representative of the total study population in the intervention study (IDA) and the nationwide DD2 cohort (Supplementary Material 3), which should be generalizable to Danish individuals with newly diagnosed type 2 diabetes (15). Another strength of this study is the use of objective and state-of-the-art physical activity classification algorithms, which bypass cognitive and social desirability biases. However, a potential limitation of this study is the use of internally validated physical activity intensity cut points. Even though cut points derive from an age-matched population, this may hamper a direct comparison to other studies. Another limitation is the accelerometers’ inherent constraints of being unable to detect weight-bearing activities of daily living or strength training. Another inherent constraint is possible misclassification of sedentary behaviors. Even though 24-h dual monitor accelerometry has qualified the measure of sedentary time due to detection of human posture, misclassification between lying awake and sleep still exists due to difficulties in detecting micro-movements.

The observed physical activity may have been influenced by participation in health education programs early after type 2 diabetes diagnosis. In Denmark, it is the responsibility of the municipalities to offer all patients an opportunity to participate in health education and lifestyle intervention programs, either group-based or individual, yet with varying content and quality, depending on each municipality. Of concern, only about 3 out of 10 individuals with newly diagnosed type 2 diabetes end up participating in the health education and lifestyle programs provided by the municipalities in Denmark (Danish Diabetes Association, personal communication). Some effect of structured lifestyle intervention therefore might be present in our study, but the majority will not have engaged in such structured activity. Further, musculoskeletal problems may have influenced our study participants’ physical activity level. Unfortunately, we do not have individual-level information on neither lifestyle intervention nor musculoskeletal problems, as this would require detailed data from the municipality of residence of each study participant, whereas mild musculoskeletal problems are usually treated in the primary care sector (e.g., physiotherapists, chiropractors, osteopaths etc.), with diagnoses and therapies not captured by our registries.

In conclusion, this study emphasizes a duality in physical activity behaviors among individuals with newly diagnosed type 2 diabetes, where recommended levels of moderate-to-vigorous physical activity can co-exist with high levels of sedentary time. Future investigations should therefore study the consequences of high sedentary time and different physical activity patterns in relation to glucose metabolism, mortality and complications in individuals with newly diagnosed type 2 diabetes. At the moment, individuals of older age, with higher BMI, with comorbidities, who are unemployed, retired or current smokers may have an increased propensity for an inactive lifestyle. Hence, clinicians should refrain from only focusing on moderate-to-vigorous physical activity, because this may be an unfeasible target for these individuals. Instead, we recommend including step count as an easy-to-communicate target (32, 33) and promoting light activities, so that more individuals are able to adhere to physical activity guidelines and reduce their total sedentary time.

## Data Availability

The datasets presented in this article are not readily available because they contain data that are owned by The Danish Health Data Authority, which cannot be made publicly available or shared by third parties due to Danish data protection legislation. Own data can be made available upon reasonable request. Requests to access the datasets should be directed to Sidsel Louise Domazet, sidsel.louise.domazet@rsyd.dk.
