# Supplementary material for: Accelerometer-derived physical activity and sedentary behaviors in individuals with newly diagnosed type 2 diabetes: A cross-sectional study from the Danish nationwide DD2 cohort
Source: Front Sports Act Living. 2023 Jan 25;4:1089579. doi: 10.3389/fspor.2022.1089579 (PMC9905636; doi:10.3389/fspor.2022.1089579)
Supplement: Supplementary file 1 [file Table1.docx]

Supplementary Material 1 – Age-specific cut points for classification of physical activity intensities using an internally conducted calibration study in middle-aged and older adults

| Age (years) | Sedentary | Light | Moderate |
| --- | --- | --- | --- |
| <60 | <100 | >100 – 3,554 | ≥3,554 |
| 60-69 | <100 | >100 - 3,409 | ≥3,409 |
| 70-79 | <100 | >100 - 2,890 | ≥2,890 |
| ≥80 | <100 | >100 - 2,516 | ≥2,516 |

| Age (years) | Vigorous |
| --- | --- |
| <70 | ≥6,332 |
| ≥70 | ≥6,225 |

Note: Cut point are Actigraph counts per minute. Moderate; equals walking at approx. 4 km/h. Vigorous; equals running equivalent to 60% of VO_2_ max.

Supplementary Material 2 – Age and education level of study participants stratified by smoking status, comorbidities and BMI

|  | Age | P-value | Education level (%) | | | P-value |
| --- | --- | --- | --- | --- | --- | --- |
|  | Median (IQI) |  | Short | Medium | Long |  |
| Smoking |  |  |  |  |  |  |
| Never | 60.7 (51.2; 68.6) |  | 39.5 | 52.9 | 7.6 |  |
| Former | 64.1 (56.2; 69.3) |  | 43.4 | 50.1 | 6.5 |  |
| Current | 59.5 (52.3; 65.6) | <0.01 | 54.7 | 42.4 | 2.9 | 0.01 |
| CCI |  |  |  |  |  |  |
| 0 | 60.1 (52.1; 67.0) |  | 41.7 | 51.2 | 7.1 |  |
| 1-2 | 64.8 (58.2; 70.3) |  | 48.2 | 48.2 | 3.6 |  |
| 2+ | 70.0 (66.2; 74.2) | <0.01 | 53.3 | 36.7 | 10.0 | 0.12 |
| BMI |  |  |  |  |  |  |
| <25 | 63.7 (59.4; 66.9) |  | 34.4 | 48.4 | 17.2 |  |
| 25-29.9 | 63.8 (55.5; 70.2) |  | 41.7 | 52.8 | 5.5 |  |
| 30-39.9 | 61.6 (53.2; 68.5) |  | 46.0 | 48.9 | 5.1 |  |
| 40+ | 56.6 (47.5; 62.0) | <0.01 | 48.7 | 46.0 | 5.3 | 0.04 |

Note: Differences were tested with Kruskal-Wallis test. CCI; Charlson Comorbidity Index.

Supplementary Material 3 – Comparison of clinical characteristics of the DD2 cohort, IDA cohort and the final study sample

|  | DD2 (total) | IDA (total) | IDA (study sample) |
| --- | --- | --- | --- |
| N | 8,198 | 1172 | 768 |
| Females, % | 41.3 | 40.8 | 42.1 |
| Age, years | 61.7 (52.8; 68.6) | 62.1 (53.7; 68.8) | 61.8 (53.7; 68.5) |
| Diabetes duration, years | 1.3 (0.3; 3.0) | 3.2 (0.7; 5.5) | 3.5 (0.9; 5.9) |
| Height, cm | 173 (165; 179) | 172 (165; 178) | 172 (165; 178) |
| Weight, kg | 91.0 (79.0; 105.0) | 92.0 (80.0; 105.8) | 91.0 (80.1; 105.2) |
| BMI, kg/m^2^ | 30.4 (27.0; 34.4) | 31.0 (27.8; 35.2) | 31.0 (28.0; 34.9) |
| Systolic BP, mmHG | 130 (124; 140) | 128 (120; 136) | 127 (120; 136) |
| Diastolic BP, mmHG | 80 (74; 86) | 82 (75; 88) | 81 (75; 88) |
| HbA1c, mmol/mol | 49 (44; 50) | 49 (45; 55) | 49 (45; 55) |
| HbA1c, % | 6.6 (5.8; 6.7) | 6.6 (6.3; 7.2) | 6.6 (6.3; 7.2) |
| C-peptide, pmol/L | 1156 (859; 1561) | 1156 (891; 1533) | 1123 (862; 1504) |
| HOMA-beta, % | 93.2 (69.9; 121.7) | 85.2 (62.7; 111.3) | 83.8 (62.6; 111.7) |
| HOMA-S, % | 35.0 (25.7; 48.0) | 34.0 (25.4; 45.0) | 35.1 (26.0; 45.8) |
| LDL-cholesterol, mmol/L | 2.2 (1.7; 2.8) | 2.1 (1.7; 2.7) | 2.1 (1.6; 2.7) |
| eGFR, mL/min/1.73m^2^ |  | 88.7 (75.9; 98.2) | 88.6 (76.3; 98.2) |
| Glucose-lowering drugs, % | 75.7 | 80.2 | 79.0 |
| Insulin, % | 5.6 | 3.8 | 3.3 |
| Lipid-lowering drugs, % | 53.9 | 66.4 | 65.4 |
| Anti-hypertensive drugs, % | 61.4 | 66.4 | 64.3 |
| Comorbidities, % |  |  |  |
| 0 | 68.8 | 72.8 | 73.4 |
| 1-2 | 25.7 | 23.5 | 23.0 |
| 3+ | 5.5 | 3.8 | 3.5 |
| Smoking, % |  |  |  |
| Never | 46.4 | 40.5 | 41.1 |
| Former | 34.4 | 39.8 | 40.6 |
| Current | 19.2 | 19.6 | 18.2 |

Note: Values are medians with interquartile intervals (25^th^ and 75^th^ percentiles) or numbers with percentage. The DD2 cohort has varying numbers of observations due to multiple data sources. BP; blood pressure. eGFR; estimated glomerular filtration rate.
